# Alkali basalts and enclosed ultramafic xenoliths near Ushuaia, Tierra Del Fuego, Argentina

**DOI:** 10.1186/s40064-016-3059-7

**Published:** 2016-08-22

**Authors:** Rogelio Daniel Acevedo

**Affiliations:** CADIC, CONICET, Ushuaia, Tierra del Fuego, Argentina

**Keywords:** Alkali basalts, Ultramafic xenoliths, Tierra del Fuego

## Abstract

At the southernmost part of Tierra del Fuego a few outcrops and erratic boulders of alkali basaltic rocks with ultramafic enclaves have been studied. Alkali basalt plugs or pipes hitherto identified are scarce, and host rocks are constituted by slates that belong to Mesozoic deposition. The petrography, texture and composition of the basalt and xenoliths were investigated by petrographic microscope and electron microprobe analysis. Xenocrysts of amphibole and alkali feldspar, phenocrysts of nepheline, olivine, spinel, phlogopite and Fe–Ti minerals (10 %) and a diversity of xenoliths, mainly lherzolitic, pyroxenite and wehrlitic nodules (15 %), but also from metamorphic rocks provenance, are contained in the basalt groundmass (75 %). This finer-grained material is made up of laths or needles of plagioclase, pyroxene, opaque minerals, apatite and glass, with intersertal, hyalopilitic and pilotaxitic. Locally, rock has an even granoblastic texture. Former amygdules are filled by analcite, zeolites, sodalite and calcite. The normative classification, based on nepheline content, conclude that this rock is an alkali basalt. The chemical classification, considering immobile elements as Zr/TiO_2_ versus Nb/Y indicate an alkali basalt too and plots over the TAS diagram fall in the foidite (Na-rich or nephelinite) and basanite fields. The REE patterns are fractionated (La/Yb primitive mantle normalized is approximately 30). The K–Ar isotopic technique on individual macrocrysts gave ages of 146 ± 5 Ma (amphibole) and 127 ± 4 Ma (alkali feldspar); and K–Ar whole rock datum reported 8.3 ± 0.3 Ma. Nevertheless, fertile samples show geochemical features typical of deep derived material thus, based on the position in the actual tectonic setting, indicate that the basalt is older than its isotopic age.

## Background

Until its recent discovery, the dense sub Antarctic forest had hidden some small outcrops of a dark brecciated basalt-like rocks, irregular and amygdaloidal, containing olivine, pyroxene, amphibole, feldspar and various lithic fragments, specially ultramafic nodules. These rocks, with their inclusions and nodules, appear spread at the foothills of the Susana Mount, West of Ushuaia city, and they are the first rocks of this type ever reported for the Isla Grande de Tierra del Fuego. Some glacial blocks of erratic boulders (brought by the glacier from the Darwin Range) are composed of the same type of rocks and samples were detected spread across the coast of Peninsula Ushuaia.

## Description of the outcrops

**Fig. 1:**
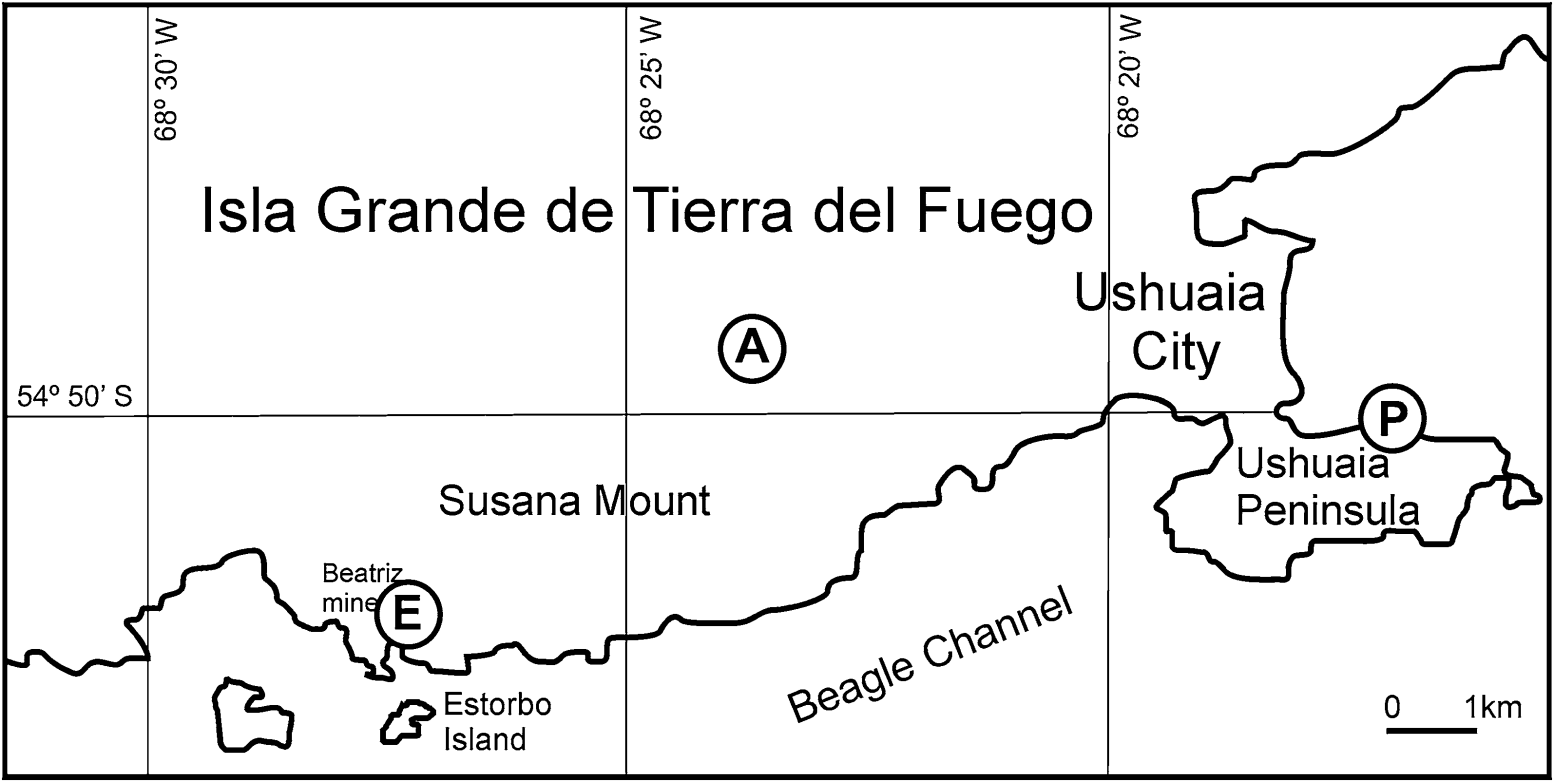
Map showing sampling locations of alkali basalts

The best exposition of the studied rocks can be observed at the base of the SW hillside of the Susana Mount, on the coast of Canal Beagle in front of the Estorbo Island, next to the Beatriz Mine, a lens of quartz and sulphides known since long time ago (Fig. 1). It consists of plugs of an amygdaloidal basalt which appear in half a dozen outcrops of small magnitude and which varies in size between 4 and 16 m. Like pipes, (samples E, Fig. 1), they spread over a reduced area (54° 51′ S; 68° 27′ W). The basalt has small and varied inclusions and its host rocks are constituted by banded gray slates which strike N75°E and dip 50/60° SE. They are attributed to the Yahgan Formation, attributed to the Upper Jurassic-Lower Cretacic age. The local geology of the area was mapped in detail in Olivero et al. (1997). A second site, close to the first one above mentioned, and next to the Ushuaia motor speedway, offers some small outcrops of the same basalt (samples A1 and A4). They are isolated, without visible contact with any surrounding rock and they contain inclusions of a larger size and macrocrysts of amphibole (sample A2) and feldspar (sample A3).

The rest of the obtained samples come from some glacial erratic blocks which are spread on the coast of Ushuaia peninsula (samples P). Later it will be displayed how these last rocks can be compared with those that are exposed in situ.

From the last statement it can be inferred that some time ago these alkali basalts were exposed on a larger area and they were later reduced to the only above mentioned isolated outcrops by the glacial erosion. Moreover, those erratic blocks are testimony of the larger original extension of the basalts and they encourage the search for new outcrops in the area.

## Petrography

Several hand specimens of and many thin sections were studied under the polarizing microscope and using the electron microprobe CAMEBAX/CAMECA of the Oviedo University, Spain (working conditions: 15 kV accelerating voltage, 20 nA beam current) more than 200 analyses were made.

The basaltic rock consists of a dark gray aphanitic matrix composed of basic glass, olivine (Fo_82–87_), clinopyroxene (augite), alkali feldspar (Ab_79–86_ An_2–6_ Or_12–15_), apatite and opaque mineral, with microtextures that go from intersertal to hyalopilitic or pilotaxitic. There are amygdales filled with analcime, natrolite and sodalite associated to calcite, this last frequently of a globular structure.

**Fig. 2:**
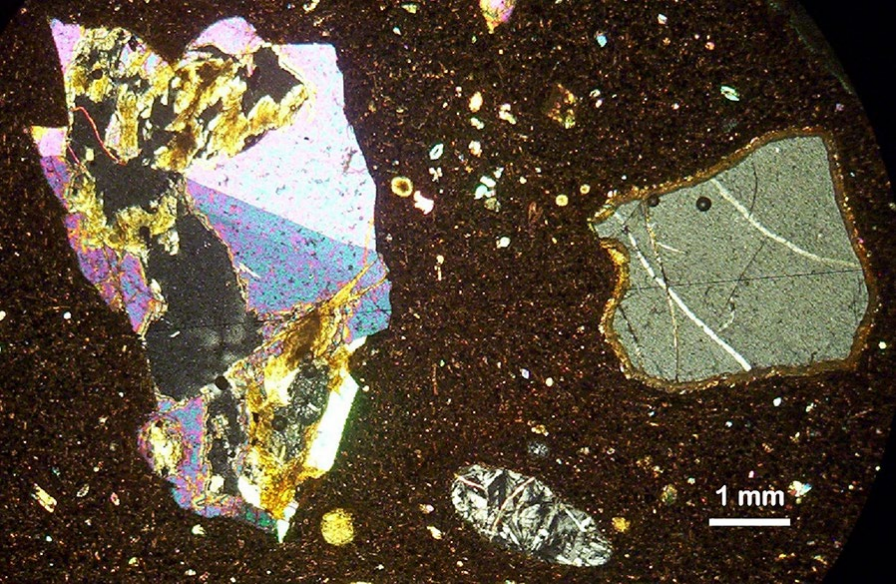
Microscopic view taken under crossed polars showing calcite in association with phlogopite and zeolites filling an amygdala and a phenocryst of nepheline surrounded by a secondary rim in a fine-grained granular groundmass

Also, the basalt has xenocrysts and xenoliths (Fig. 2). The xenocrysts have rounded shapes (as a consequence of the instability in the melted magma) and they present varied sizes and appearances. Among them there are specimens of amphibole (kaersutite) and Na–K alkali feldspar which are of a larger size that the rest of them. Scarce clinopyroxene (En_53_ Fs_11_ Wo_36_) and orthopyroxene (En_87-92_) xenocrysts were also identified. Other individuals are spinels (surrounded by reaction rims of magnetite or ilmenite), magnetite-ulvospinel, phlogopite and crystals of olivine (Fo_81–91_), sometimes in groups.

Among the numerous and varied enclaves, in addition to the sedimentary and metamorphic (slates, quartzites and gneisses) lithic fragments, many belong to ultramafic rocks, peridotites and pyroxenites (clinopyroxenites and websterites), some lherzolites, wehrlites and also gabbros. All of them are in a quite advanced state of deformation (deformation banding) and/or recrystallization (granoblastic texture). Specifically, an ultrabasic nodule contains titaniferous garnet (pyrope), clinopyroxene and bytownite (An_85_Ab_15_).

## Geochemistry

A set of ten samples of whole rock ICP analysis of major elements was performed using 0.2 g each specimen, fused with 1.5 g of LiBO_2_ and dissolved in 100 mls 5 % HNO_3_. Five samples of them, deliberately selected, were analyzed by REE using LiBO_2_ fusion, ICP/MS finished.

The normative classification, based on the nepheline’s content (ab + an + or + ne + di + ol), presents this rock as an alkali basalt (sub-alkali for sample E1). The chemical classification, taking in account the relation Zr/TiO_2_ versus Nb/Y in altered rocks (Winchester and Floyd 1977), also indicated an alkali basalt (even for sample E1), and its projection in the TAS diagram shows it as a foidite (Na-rich or nepheline) or a basanite, depending of their silica content (by itself very depleted) although sometimes the mentioned depletion of silica (a good indication of ultrabasicity) appears exaggerated due to its degree of alteration, its content of volatiles and the loss by ignition in the analytical handling of the samples as can be seen in the attached analysis (Table 1). In that sense, sample E1 (Ab_61_ An_38_) could be taken as a hawaiite.

**Table 1:** Major element oxide compositions (wt %) of fuegian alkali basalts *Source*: D. Toye, C. Leong, J. Wang (ACME Labs)

	SiO_2_	TiO_2_	Al_2_O_3_	Fe_2_O_3_	MnO	MgO	CaO	Na_2_O	K_2_O	P_2_O_5_	LOI
P1	37.15	2.39	11.52	13.07	0.29	9.02	12.02	2.84	2.05	2.53	6.60
P2	38.95	2.51	11.75	12.76	0.25	9.79	11.51	3.21	1.42	2.01	5.40
P 3	43.90	2.61	13.19	12.46	0.23	6.27	8.96	4.03	2.27	1.36	3.80
P 22	41.04	2.47	12.23	13.39	0.23	9.19	9.90	4.18	0.95	1.63	3.89
P V-01	37.87	2.38	11.66	14.08	0.28	7.96	12.65	2.81	2.24	2.57	3.44
A 1	40.25	2.70	12.91	12.91	0.23	7.42	10.09	2.86	1.94	1.35	7.00
A 2	40.83	2.81	12.77	14.45	0.23	6.96	10.47	2.20	2.49	1.60	3.90
A TF-10	40.52	2.76	12.73	14.29	0.22	6.87	10.35	2.23	2.58	1.57	4.23
A TF-12	40.40	2.75	12.69	14.27	0.23	6.95	10.62	2.07	2.30	1.53	4.26
E 1	43.63	2.80	13.53	13.04	0.24	4.83	8.27	3.32	1.05	1.36	7.40

**Fig. 3:**
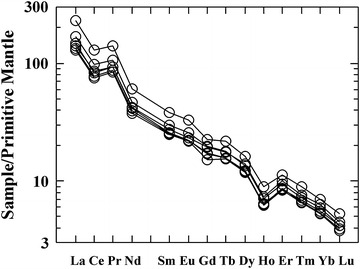
REE content of samples normalized to that of the primitive mantle, showing elevated LREE and depressed HREE. *Source*: D. Toye, C. Leong, J. Wang (ACME Labs)

Chemical analysis of Rare Earth Elements (REE) was also preformed to give an additional aspect of comparison between the different rock samples, mainly with those obtained from the erratics. The result was a very exact match in the data, very remarkable, of the values normalized to the primitive mantle (Fig. 3). The graphs show a high content in Light Rare Earth Elements (LREE) and depressed values of Heavy Rare Earth Elements (HREE), indicating its high degree of fractionation (La/Yb ~ 30) and subsaturation.

## Age

Three K/Ar isotopic ages were performed, one of them on the whole rock and two on macrocrysts of amphibole and alkali feldspar, respectively. The amount of Ar was measured by mass spectrometry of the gases released when the sample was melted in vacuum. The potassium was quantified by atomic absorption spectroscopy.

The intention of these isotopic datings was to establish, in an approximate way, the possible chronological difference between the crystals and the pure mass of the rock (Table 2).

**Table 2:** Isotopic datings of xenocrysts and alkali basalt *Source*: E. Linares (INGEIS)

Sample	K %	^40^K = ×10^−8^mol/g	^40^Ar_rad_ = ×10^−10^mol/g	^40^Ar_atm_ = %	Age Ma	Analytical error = %
AK 4133 amphibole	1.75	5224	4617	79.10	146 ± 5	3.40
AK 4134 alkali feldspar	2.54	7582	5818	70.80	127 ± 4	3.50
AK 4137 whole rock	2.04	6089	0.296	91.10	8.30 ± 0.30	2.90

## Conclusions

Despite the limited field information, it is necessary to learn about the age of the eruptive event. The resulting ages found for the xenocrysts were coherent. The older age obtained for the amphibole (AK-4133) is explained by its larger retentivity of argon in regard to the analyzed alkali feldspar (AK-4134). Moreover, the feldspar has a potassium content of 2.54 % which reveals the presence of acid feldespar, which also opens the possibility (due to its type of structure) of a loss in argon. In synthesis, the ages of 147 and 126 Ma (Tithonian–Neocomian) for the xenocrysts could be taken as real, being the older one the more accurate. On the other hand, the extraction in the basalt sample (AK-4137), although the high content of 40Ar_atm_ (91.1 %), would indicate a young rock but that result is inconclusive (Linares, private communication, 1997).

Unfortunately, the isotopic age determinations data are not conclusive. It is clear that the xenocrysts are old, probably from lower Cretaceous, which gives evidence of a great difference in time with the age obtained for the rock. The age of the basalt is the quid of the question. It could be theoretically placed, by the local geology, in the Mesozoic times. In Tierra del Fuego, there have been reports of basalts, although not alkali, in the Mesozoic formations but their characteristics are quite different (Stern et al. 1985; Acevedo 1988, 2004; Olivero et al. 1997; Martinioni et al. 1999; Acevedo and Quartino 2004; Seraphim et al. 2005). The more distant Pliocene-Quaternary basalts of Pali Aike in Santa Cruz Province are in fact alkaline (Stern et al. 1985; Kempton et al. 1999a, b).

As stated, the basalt studied here gives an isotopic age (by the K/Ar method on the whole rock) that corresponds to the upper Miocene but a younger age could not be ignored (Linares, private communication, 1997). Even doing abstraction of the data related to the Patagonian olivine basalts, it would keep its real value (Kilian et al. 1998). In that case, it would be another example derived from the Patagonian olivinic basalt volcanism (Laurora et al. 2001). Particularly, the olivine basaltic magma (the one that moved upward in the times of the distention of the foreland) is typical of the extrandine Patagonia and has two main characteristics: its regional extension and its capacity to generate alkali basalts, its classic site being the Sierra de San Bernardo in Chubut Province (Mórtola 1923; Quartino 1958; Teruggi 1963; Viviers 1968, 1970) and another places in Patagonia (Aliani et al. 2004). Those characteristics extend to other Patagonian areas and, in close connection with this work, to the south of Santa Cruz Province in front of the fuegian coasts.

The enclosed ultramafic inclusions speak of a deep origin, in connection with the mantle. Kaersutitic compositions are common in mantle but also in volcanic-arc environments (Gentili et al. 2015). The mantle’s procedence is supported by the presence of nodules with the typical composition and textures of the upper mantle. They were incorporated to the magma in depth and ended in the alkali basalt outcrops.

Those ultramafic xenoliths reflect a subcontinental procedence (they are very fertile) and as they are not refractory, they cannot come from a suprasubduction zone (Nelson 1996). This last statement would be indirect evidence to reject the hypothesis that the alkali basalts of Tierra del Fuego could belong to a recent eruptive event.
